# Complete genome sequence of an Israeli isolate of *Xanthomonas hortorum* pv. pelargonii strain 305 and novel type III effectors identified in *Xanthomonas*

**DOI:** 10.3389/fpls.2023.1155341

**Published:** 2023-06-02

**Authors:** Naama Wagner, Daniella Ben-Meir, Doron Teper, Tal Pupko

**Affiliations:** ^1^ The Shmunis School of Biomedicine and Cancer Research, George S. Wise Faculty of Life Sciences, Tel Aviv University, Tel Aviv, Israel; ^2^ Department of Plant Pathology and Weed Research, Institute of Plant Protection Agricultural Research Organization (ARO), Volcani Institute, Rishon LeZion, Israel

**Keywords:** Xanthomonas, type-III secretion system, Effector proteins, type-III effectors, machine learning, Effectidor

## Abstract

*Xanthomonas hortorum* pv. pelargonii is the causative agent of bacterial blight in geranium ornamental plants, the most threatening bacterial disease of this plant worldwide. *Xanthomonas fragariae* is the causative agent of angular leaf spot in strawberries, where it poses a significant threat to the strawberry industry. Both pathogens rely on the type III secretion system and the translocation of effector proteins into the plant cells for their pathogenicity. Effectidor is a freely available web server we have previously developed for the prediction of type III effectors in bacterial genomes. Following a complete genome sequencing and assembly of an Israeli isolate of *Xanthomonas hortorum* pv. pelargonii - strain 305, we used Effectidor to predict effector encoding genes both in this newly sequenced genome, and in *X. fragariae* strain Fap21, and validated its predictions experimentally. Four and two genes in *X. hortorum* and *X. fragariae*, respectively, contained an active translocation signal that allowed the translocation of the reporter AvrBs2 that induced the hypersensitive response in pepper leaves, and are thus considered validated novel effectors. These newly validated effectors are XopBB, XopBC, XopBD, XopBE, XopBF, and XopBG.

## 1 Introduction

The *Xanthomonas* genus includes dozens of species divided to thousands of subspecies and strains with a wide range of lifestyles: from commensal, to opportunistic, to pathogenic. Among them are some of the major plant pathogens worldwide, affecting more than 400 plant species (Timilsina et al., 2020). These pathogens rely on the type III secretion system (T3SS) and type III effectors (T3Es) for their pathogenicity. The effectors alter processes within the host cell for the benefit of the bacteria and thus promote disease in the plant (White et al., 2009; Ryan et al., 2011; An et al., 2019). Identification of the full effector repertoire encoded within the genome of a pathogenic bacterium is a prerequisite for detailed understanding of the molecular interactions between the pathogen and its host.

Discovering novel effectors is a challenging task, as effectors are highly diverse in their functionality, size, and structure. Moreover, the effector repertoire varies even among closely related strains (Jalan et al., 2013; Jiménez-Guerrero et al., 2020). The T3SS recognizes T3Es based on a secretion signal located in their N-terminus (Michiels and Cornelis, 1991; Sory and Cornelis, 1994). However, despite extensive efforts to characterize it, the secretion signal of T3Es is not characterized enough to allow accurate prediction of effectors as a sole feature (Wagner et al., 2022a). We have previously developed and applied machine-learning techniques to identify T3Es and type IVb effectors in various pathogenic bacteria (Burstein et al., 2009; Lifshitz et al., 2013; Lifshitz et al., 2014; Burstein et al., 2015; Burstein et al., 2016; Teper et al., 2016; Nissan et al., 2018; Jiménez-Guerrero et al., 2020; Ruano-Gallego et al., 2021). Following these efforts, we developed Effectidor: an automated machine-learning based web server for the prediction of T3Es (Wagner et al., 2022b). Effectidor combines dozens of different features to achieve accurate classification, e.g., sequence similarity to previously validated effectors, sequence similarity to host proteins, and atypical GC-content. Another feature is the sequence similarity to closely related bacteria without T3SS (putative effectors are expected not to have strong hits when searching against such genomes). Additional features that we consider are the amino acid composition, the genomic organization (effectors often reside close to each other in the genome), existence of known regulatory elements that are recognized by transcriptional regulators that regulate the T3SS and some of the T3Es, such as the plant-inducible promoter (PIP)-BOX (Cunnac et al., 2004; Koebnik et al., 2006), and a signal score reflecting the likelihood of the existence of a secretion signal in the 100 N-terminal amino-acids of the protein (Wagner et al., 2022a). Using these features, Effectidor trains a machine-learning classifier on the known effectors and non-effectors of the specific bacterial genome it analyzes, and outputs a prediction for all the other protein coding genes in the genome, reflecting their likelihood to encode an effector. Thus, we can pinpoint the T3Es candidates in the genome with no need for labor and cost intensive full-genomic screening. In this work we applied Effectidor to two *Xanthomonas* pathogens: *Xanthomonas fragariae*, the causative agent of angular leaf spot (ALS) in strawberries, and *X. hortorum* pv. pelargonii, the causative agent of bacterial blight in geranium.

The pathogen *X. fragariae* (Xfrg) was first reported in Minnesota, the United States in 1960 (Kennedy and King, 1962), and since then it has spread worldwide (Mazzucchi et al., 1973; McGechan and Fahy, 1976; Gubler et al., 2007; Matthews-Berry and Reed, 2009; Fernández-Pavía et al., 2014; Kamangar et al., 2017; Wu et al., 2020; Song et al., 2021). This bacterium is a quarantine pathogen in Europe and it is currently widely spread in North America, where it causes substantial loss in the strawberry nursery industry (Puławska et al., 2020). In severe cases of the disease the crop production is significantly reduced either due to death of the plant or due to changes in the appearance of the fruit, which make them unmarketable. Yet, the most severe economic threat is to nurseries, where the bacteria spread easily. Currently there are neither resistant strawberry plants, nor effective treatments against the pathogen (Wang et al., 2018).

The bacterium *X. hortorum* pv. pelargonii (Xhp) is the causal agent of bacterial blight in geranium ornamental plants (also known by the name “pelargonium”). This is the most threatening bacterial disease of these plants worldwide (Barel et al., 2015; Balaž et al., 2016). The disease is widespread in various states of the USA, Europe, Australia and Israel, and may cause heavy economic losses. Warm and wet conditions favor infection and disease development. Normally, Xhp penetrates the plant *via* natural openings or wounds, and spreads systemically through the vascular system. Symptoms are characterized by wilting of the plant, localized water-soaked lesions that often become necrotic and rotted cuttings. All commercial cultivars of geranium are susceptible to Xhp (Zhang et al., 2009).

In this work, we aimed to discover new T3Es in these two pathogens. We first sequenced the genome of an Israeli isolate of Xhp, combining short and long reads to obtain high quality genome sequence. Next, we applied Effectidor (Wagner et al., 2022a; Wagner et al., 2022b; Wagner et al., 2022c) to predict T3Es in these two genomes. Our results suggested the existence of unknown T3Es in both genomes, i.e., putative T3Es without significant sequence similarity to previously identified effectors. We next experimentally validated the translocation of some of these putative T3Es in a T3SS mediated manner. We validated two and four novel T3Es in Xfrg and Xhp, respectively.

## 2 Materials and methods

### 2.1 Bacterial strains and plant material

The bacterium *X. hortorum* pv. pelargonii (Xhp) strain 305 was isolated from geranium plants in Israel and was a gift from Dr. Shulamit Manulis-Sasson from the Agricultural Research Organization (ARO), Volcani Center Israel (Barel et al., 2015). Genomic DNA of *X. fragariae* (Xfrg) Fap21 (BioSamble SAMN05505397) (Henry and Leveau, 2016) was kindly provided by Dr. Joël Pothier (Zurich University of Applied Sciences). For the translocation assays, we used *X. euvesicatoria* (Xeu) hrpG* ΔavrBs2 (Roden et al., 2004). For cloning, we used NEB 5-alpha *Escherichia coli* that were obtained from New England Bio-Labs inc.

Strains of *E. coli* and *Xanthomonas* were grown in Luria–Bertani (LB), broth or agar, at 37°C and 28°C, respectively. The antibiotics used were spectinomycin (Sp; 100 μg/ml), kanamycin (Kan; 50 μg/ml) and gentamicin (Gm; 10 μg/ml). All antibiotics were from Sigma-Aldrich.

Pepper plants (*Capsicum annuum*) ECW20R (Kearney and Staskawicz, 1990) were grown in the greenhouse at 25°C and kept in long‐day conditions (16 h light, 8 h dark).

### 2.2 Genome sequencing of *X. hortorum* pv. pelargonii 305

Genomic DNA of Xhp305 was isolated from 3 ml of overnight culture using Wizard^®^ Genomic DNA Purification Kit – Promega. Microbial *De novo* sequencing was performed at Novogene Co., Ltd. using both PacBio (PacBio Sequel II) and Illumina (NovaSeq 6000) platforms. The shotgun genomic library for short-read sequencing and the library for long-read sequencing were prepared by the service provider, who also performed quality control. The Illumina sequencing yielded 17,914,078 paired-end reads of length 150 bp. The PacBio sequencing yielded, after trimming, 245,660 subreads, with mean length of 11,006 bp, N50 of 13,343 bp, for a total of 2,704 Mbp.

#### 2.2.1 Genome assembly and annotation

For *de novo* assembly, the whole set of PacBio subreads was used as input for Canu v2.2 (Koren et al., 2017) with the following parameters: -pacbio-raw genomeSize = 5.6m. The average coverage was assessed by mapping corrected and trimmed reads obtained by Canu v2.2 against the assembly using BWA v0.7.17 (Li and Durbin, 2009) with default parameter values, calculating the alignment depth using SAMtools v1.3.3 (Li et al., 2009) with default parameter values, and the average depth per molecule using awk. We then used the draft genome and the corrected PacBio reads as input for Circlator (Hunt et al., 2015), together with BWA v0.7.17 (Li and Durbin, 2009), prodigal v2.6.3 (Hyatt et al., 2010), SAMtools v1.3.3 (Li et al., 2009), MUMmer v3.23 (Kurtz et al., 2004), and Canu v2.2 (Koren et al., 2017) to circularize the chromosome and plasmids, with the following parameters: circlator all –assembler canu. Following this step, we used the Illumina reads to polish the assembly using Pilon v1.22 (Walker et al., 2014), BWA v0.7.17 (Li and Durbin, 2009) and SAMtools v1.3.3 (Li et al., 2009) with the default parameter values and including –changes to keep track of the corrections done in the assembly. We applied three rounds of polishing using Pilon, until no further corrections were introduced in the fourth round. The average coverage of the Illumina reads was assessed in the same manner as assessed for the PacBio reads. For genome annotation, we used Prokka v1.13.3 (Seemann, 2014) with default parameter values.

### 2.3 Effectors prediction

Effectidor v1.04 (Wagner et al., 2022b) was used for T3Es predictions in each of the two genomes. The pipeline within Effectidor is divided into the following steps: (1) Defining the positive T3Es in the input genome either based on the input supplied by the user or based on homology to previously validated T3Es from various strains (this dataset can be viewed and downloaded from https://effectidor.tau.ac.il/data.html. For the analysis done in this work, version 1.04 was used). The homology criteria are E-values smaller than 10^-10^ and at least 70% identical matches. If less than five effectors are identified based on this cutoff, the last criterion is reduced by 10 (i.e., 60% identical matches are required instead of 70%) until a minimum of 40% identical matches; (2) Defining the negative set (i.e., non T3Es encoded in the input genome) based on homology to proteins of *E. coli* K12 MG1655 (accession GCF_000005845.2); (3) Feature extraction. The features used in Effectidor vary based on the provided input. While the only mandatory input in Effectidor is a FASTA file containing all the ORFs records in the genome, additional inputs allow extraction of features outside the gene sequence alone. In our analysis we provided the following additional inputs, available in the advanced options of Effectidor: (3.1) GFF file, which holds information about the location of all the genes in the genome and allows Effectidor to extract genome organization features; (3.2) FASTA file of the full genome, which allows, together with the GFF file, to search for the PIP-box regulatory element in the promoters of the genes; (3.3) ZIP archive with FASTA files holding protein records of *Luteimonas* sp. MC1825 (accession GCF_014764385), *Lysobacter capsica* 55 [accession GCF_001442785 (de Bruijn et al., 2015)], *Pseudoxanthomonas suwonensis* J1 [accession GCF_000972865 (Hou et al., 2015)], *Stenotrophomonas maltophilia* K279a [accession GCF_000072485 (Crossman et al., 2008)], and *Xylella fastidiosa* 9a5c [accession GCF_000006725 (Simpson et al., 2000; Marques et al., 2001)] that were used as input for the proteomes of closely related bacteria without T3SS. This input allows to run homology searches against these proteomes, and the results of these searches often serve as informative features for the machine-learning classifier, as T3Es are not expected to be found in these genomes, whereas many of the non-T3Es – are. In addition to these inputs, we conducted all searches including the optional feature that predicts the presence of the type 3 secretion signal in the protein sequence (Wagner et al., 2022a); (4) Training a machine learning classifier. Following the feature extraction step, several classifiers (i.e., Linear Discriminant Analysis, Naïve Bayes, Support Vector Machine, Logistic Regression, K Nearest Neighbors, and Random Forest) are trained on the labeled data (i.e., T3Es and non-T3Es defined in the first step). The labeled data are split into train and test sets, the classifiers are first trained in cross-validation on the training set (including feature selection) and are finally evaluated on the test set. The evaluation method used in Effectidor is the Area Under the Precision-Recall Curve (AUPRC). The temporary best classifier is defined as the one with the highest AUPRC on the test set. All classifiers are then evaluated according to the following criteria: (4.1) AUPRC measured on the test set is smaller than that achieved by the temporary best classifier by no more than 30%; (4.2) The range of the prediction scores of the genes in the training set is at least 0.75, to ensure that not all samples are classified as negative/positive; (4.3) The AUPRC measured on the train set and on the test set are compared, and the difference must be smaller than 0.25, to reduce chances of overfitting. The classifiers that meet all these criteria, are then merged to form a final voting classifier; (5) Applying the final classifier to identify potential novel T3Es in the genome. The final voting classifier is then applied to produce a score between 0 and 1 for each ORF in the genome. This score reflects the likelihood for this ORF to encode a T3E. Of note, in case all classifiers were dropped for not meeting some of the criteria mentioned in (4.3), the final classifier used to produce these predictions is a vote over all classifiers. In this case a message is sent to the user of Effectidor. Genomes with a small number of known effectors are more susceptible to it.

### 2.4 Translocation assay

#### 2.4.1 Plasmid construction

The plasmid pAvrBs2‐HR (KanR) containing the Hypersensitive Response (HR) domain of *avrBs2* (amino acids 62–574), fused to an haemagglutinin (HA) tag (Teper et al., 2016), was used as vector for cloning and expression of candidate effector genes. The vector was linearized with XhoI and XbaI restriction enzymes (Thermo Fisher Scientific). The putative T3E genes of Xhp and Xfrg, including 24 bp upstream of their ATG start codon, were PCR amplified (Phusion Hot Start II High-Fidelity DNA Polymerase, Thermo Scientific) from genomic DNA of Xhp305 and XfrgFap21 using gene specific primers (**Supplementary Table S1**). In most cases the whole candidate gene was amplified, but in two cases, (PML25_02815 and PML25_02835) where the suspected gene was extremely long (> 3,000 bp), only the first ~600 bp were amplified. PCR products were purified and assembled (Gibson Assembly^®^ Cloning Kit, NEB) into the linearized pAvrBs2‐HR vector, upstream to the HR domain, according to the manufacturer directions. Assembly products were initially transformed into NEB 5-alpha competent *E. coli* according to the kit’s instructions and grown on LB-Kan plates. The plasmids were then mobilized into Xeu hrpG*Δ*avrBs2* that constitutively expresses the T3S apparatus and contains a mutation in the *avrBs2* gene, by using pRK2013 as a helper plasmid in triparental mating, as previously described (Figurski and Helinski, 1979). Conjugants were selected on LB-Kan-Gm plates. Presence of the recombinant plasmid was verified in each conjugated recipient by colony PCR using insert specific primers and by Sanger sequencing using the same primers.

#### 2.4.2 Translocation

For translocation assays (Roden et al., 2004), overnight bacterial cultures were suspended in 10 mM MgCl_2_ at an optical density of 0.1 (at 600 nm) and infiltrated into the leaves of 7‐week‐old ECW20R (carrying the Bs2 gene) pepper using a needleless syringe. Elicitation of HR was monitored at 36 h post‐inoculation. For visualization of cell death, leaves were harvested and soaked for 24 h in a bleaching solution (40% ethanol, 40% chloroform, 10% acetic acid), and then transferred to a recovery solution (40% glycerol, 10% ethanol). For each translocation assay, three leaves of at least three pepper plants were infiltrated. Experiments were repeated three times with similar results.

## 3 Results

### 3.1 Genome assembly of *X. hortorum* pv. pelargonii strain 305

The genome assembly of Xhp305 was carried out using both long (PacBio Sequel II) and short (Illumina NovaSeq 6000) reads. Using the long reads of PacBio we could close the circular genome, and the short Illumina reads were used to polish the assembly. The assembly resulted with three circular molecules: a chromosome of 5,216,813 bp, and two plasmids of 188,317 bp, and 51,091 bp, with average coverage of 36X, 35X, and 54X, respectively. The assembly polishing using the Illumina reads resulted with a few corrections in the chromosome and in the smaller plasmid. The average coverage of the assembly measured by mapping the Illumina reads was 454X, 467X, and 1,674X for the chromosome and plasmids, respectively. The coverage of the plasmids relative to the chromosome, measured both using the PacBio and the Illumina reads, suggests that the larger plasmid has a copy number of one, while the copy number of the smaller plasmid is either two or three, depending on the coverage of the PacBio versus Illumina, respectively. The average GC content of the chromosome is 0.64, while the average GC contents of the larger and smaller plasmids are 0.59 and 0.62, respectively. The chromosome and plasmids hold 4,357, 191, and 62 coding sequences, respectively. Genome assembly features are available in **Table 1**. The sequencing data and assembled genome were deposited to NCBI and can be found in BioProject PRJNA926924. The annotation available in NCBI was done using the internal PGAP annotation pipeline of NCBI. It differs from the annotation we obtained using Prokka, mainly in the prediction of translation start sites. The genomic features estimated here are based on the Prokka annotation. Our downstream analysis was done using Prokka annotation, and this annotation is available in the supplementary data.

**Table 1:** Features of the assembled Xhp305 genome following sequencing with PacBio and Illumina, assembly with Canu, polish with Pilon, and annotation with Prokka.

Feature	Chromosome	plasmid1	plasmid2
**Size (bp)**	5,216,813	188,317	51,091
**No. of circular contigs**	1	1	1
**No. of CDSs**	4,357	191	62
**G+C content (%)**	63.8	58.8	62.1
**Pac-bio average coverage**	36X	35X	54X
**Illumina average coverage**	454X	467X	1,674X
**No. of tRNA genes**	54	1	0
**No. of T3Es (+ No. of T3Es that were validated here)**	35(+4)	0	1(+1)

#### 3.1.1 Detection of recent transposon duplication

**Figure 1:**
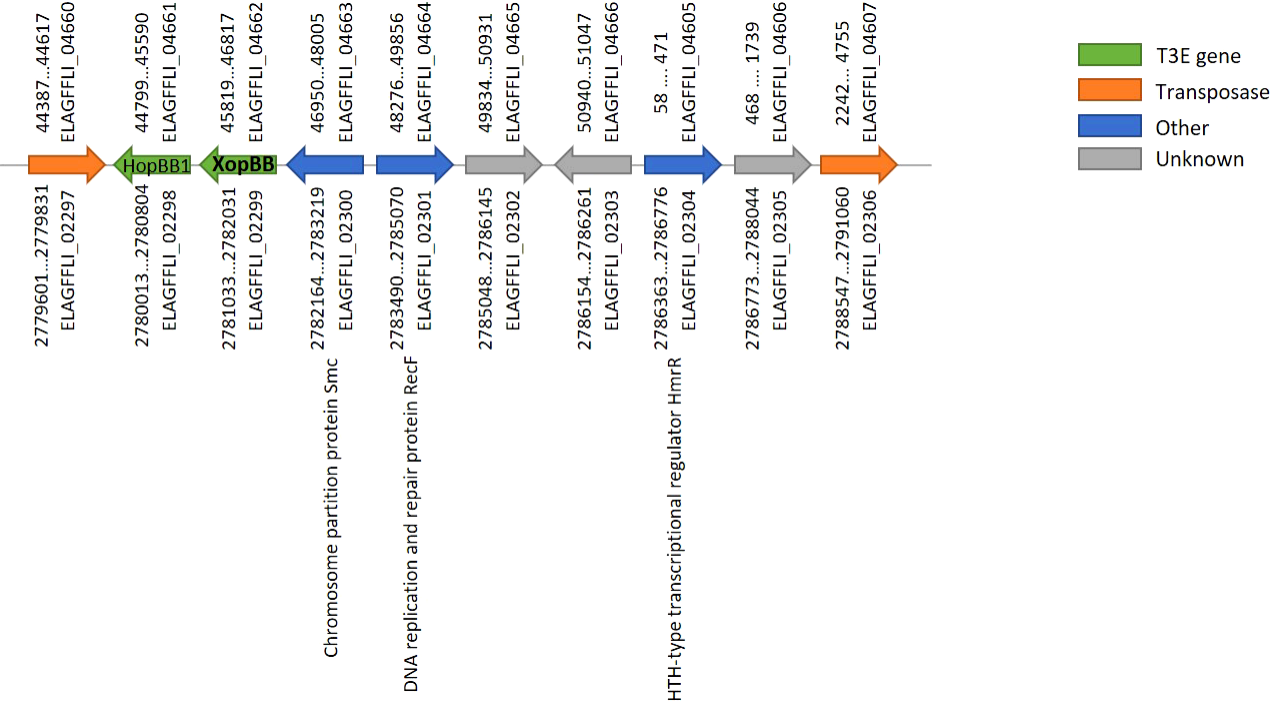
Gene order on the transposon found both on the chromosome and plasmid of Xhp305. The locus tags above represent ORFs on the plasmid, and below represent ORFs on the chromosome. ELAGFFLI_02299/ELAGFFLI_04662 was validated here as a T3E, named XopBB.

Interestingly, an identical DNA segment of 11,818 bp, containing ten ORFs (see **Supplementary Table S2**), was found both on the chromosome and on the smaller plasmid. This segment is identical by DNA sequence based on the PacBio assembly, and no corrections were introduced within it using the Illumina reads mapping. Based on Prokka annotation, the first and last ORFs in this segment encode for two DNA transposases (IS3 family transposase ISMex7 and Tn3 family transposase ISPa43), which explains this duplication (termed transposon from now on). In order to verify the location of the two identical transposons (one chromosomal and one on the plasmid) we performed several PCR reactions on the total DNA prep we had previously sent for sequencing: Primers were designed to produce ~900 bp products covering the junction points between the chromosome/transposon or the plasmid/transposon (see primers table in the supplementary data), assuming that no product would be obtained in the plasmid/transposon combination if the transposon only existed on the chromosome and vice versa. Results of the PCR reaction (**Figure S1**) clearly show that the identical transposon can be found both on the chromosome and on the plasmid. The PCR products were Sanger-sequenced and found to be identical to the sequences of their respective source; chromosome or plasmid. The lack of point mutations between the two copies of the transposon indicates that the duplication event was very recent, on an evolutionary scale. A BLASTn search of the two transposases at the edges of this transposon, yielded identical hits to the chromosome of other Xhp strains. This suggests that the transposon was originally on the chromosome and was then duplicated to the plasmid of Xhp305. Among the ten ORFs found on the transposon we identified two T3Es, HopBB1 and XopBB. The latter was validated here (see below). Other than the T3Es and the transposases, according to Prokka annotation, it also holds a chromosome partition protein SMC (Strunnikov, 2006), a DNA replication and repair protein, and a HTH-type transcriptional regulator HmrR, which stands for heavy metal-responsive regulator. The order and location in the genome of the ORFs on this segment is illustrated in **Figure 1**.

#### 3.1.2 T3SS and T3Es genes on the genome

**Figure 2:**
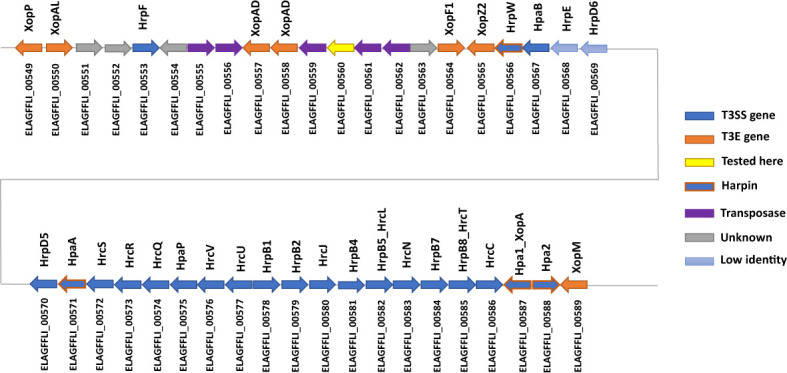
Illustration of the gene order of the hrp2 cluster and adjacent T3E genes on the chromosome of Xhp305. In blue are the hrp2 genes. In light blue are genes with low sequence similarity (less than 50% identity) to the respective hrp2 gene of Xcc. In orange are T3E genes, while genes with orange frame and blue filling are harpins. In yellow is a gene that was tested here for translocation and was not translocated.

The Xhp305 genome possesses a full hrp2 class T3SS on the chromosome, but some of these genes are with a low percentage of identical matches to the respective *X. campestris* pv. campestris (Xcc) hrp2 genes that were used as reference. Specifically, ELAGFFLI_00568 (PML25_02850) has only 35% identity with Xcc HrpE (AAM40519), and ELAGFFLI_00569 (PML25_02855) has only 45% identity with Xcc HrpD6 (AAM40520). The genes order is the same as in the reference T3SS cluster from Xcc. **Figure 2** shows the cluster of T3SS genes, adjacent T3E genes and harpins (Choi et al., 2013). Other than the two T3Es on the transposon, all the other T3Es found in this genome are on the chromosome.

#### 3.1.3 Xhp305 genome compared to other *Xanthomonas hortorum* genomes

To compare the genome of Xhp305 with other *Xanthomonas hortorum* (Xh) genomes, we downloaded all the fully sequenced genomes of Xh available in NCBI on January 10^th^ 2023, and used them, together with our genome of Xhp305, as well as Xfrg genome, as input for M1CR0B1AL1Z3R (Avram et al., 2019). M1CR0B1AL1Z3R is a web server for the analysis of large-scale microbial genomics data. We used default parameter values with the following changes: Maximal e-value cutoff of 10^-4^, Xfrg genome as an outgroup for the phylogeny reconstruction, and bootstrap over the species tree. The following genomes of Xh were analyzed: (1) Xh strain VT106 (accession GCF_008728175); (2) Xh pv. vitians LM16734 [accession GCF_014338485 (Morinière et al., 2021)]; (3) Xh pv. vitians strain CFBP498 (accession GCF_903978195 (Dia et al., 2020)); (4) Xh strain Oregano108 (accession GCF_026651895); (5) Xh strain jj2001 [accession GCF_024339125); (6) Xh pv. gardneri strain JS749-3 [accession GCF_001908755 (Richard et al., 2017)]; (7) Xh pv. gardneri strain ICMP7383 [accession GCF_001908775 (Richard et al., 2017)]; (8) Xh pv. gardneri strain CFBP8129 [accession GCF_903978225 (Dia et al., 2020)]; and (9) Xh strain B07-007 (accession GCF_002285515). The following Xhp, other than Xhp305, were also included in the analysis: (1) Xhp strain OSU778 (accession GCA_025452115); (2) Xhp strain OSU498 (accession GCA_024498995); and (3) Xhp strain OSU493 (accession GCF_024499015). Together with the strains studied here, Xhp305 and Xfrg, a total of 14 strains were used in this analysis.

**Figure 3:**
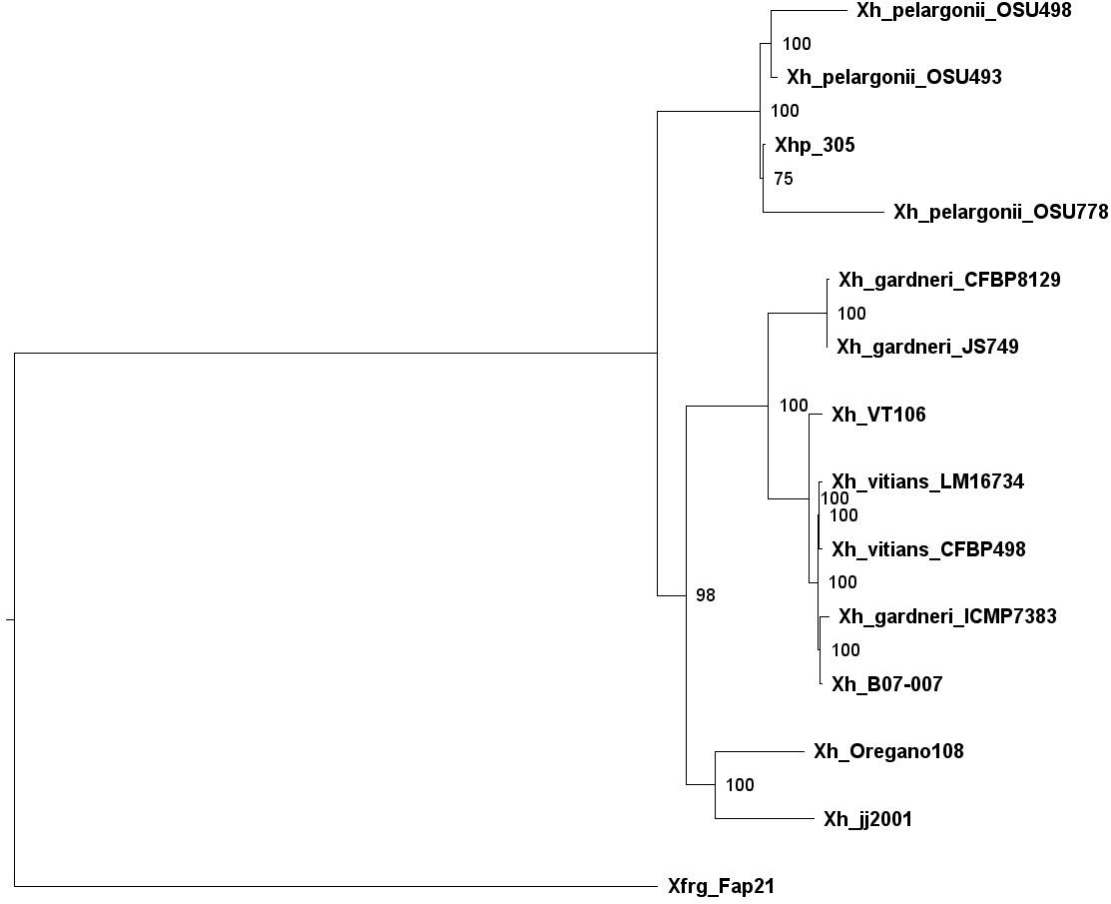
Phylogenetic tree of *Xanthomonas hortorum*, Xhp305, and Xfrg, reconstructed by M1CR0B1AL1Z3R based on the core genome of these strains.

Using a minimum identity score of 80%, M1CR0B1AL1Z3R found 5,983 orthologous groups among these 14 genomes. **Figure S2** summarizes the frequencies of the orthologous groups’ sizes. As can be seen in this figure, of these 5,983 groups, 2,350 groups included genes shared by all genomes, thus defining the core genome. Using this core genome, M1CR0B1AL1Z3R reconstructed the phylogenetic tree (**Figure 3**). Based on this tree, we infer that the Israeli isolate, Xhp305, is evolutionary close to Xhp strain OSU778, isolated from a geranium leaf sample in the USA in 2012.

**Figure 4:**
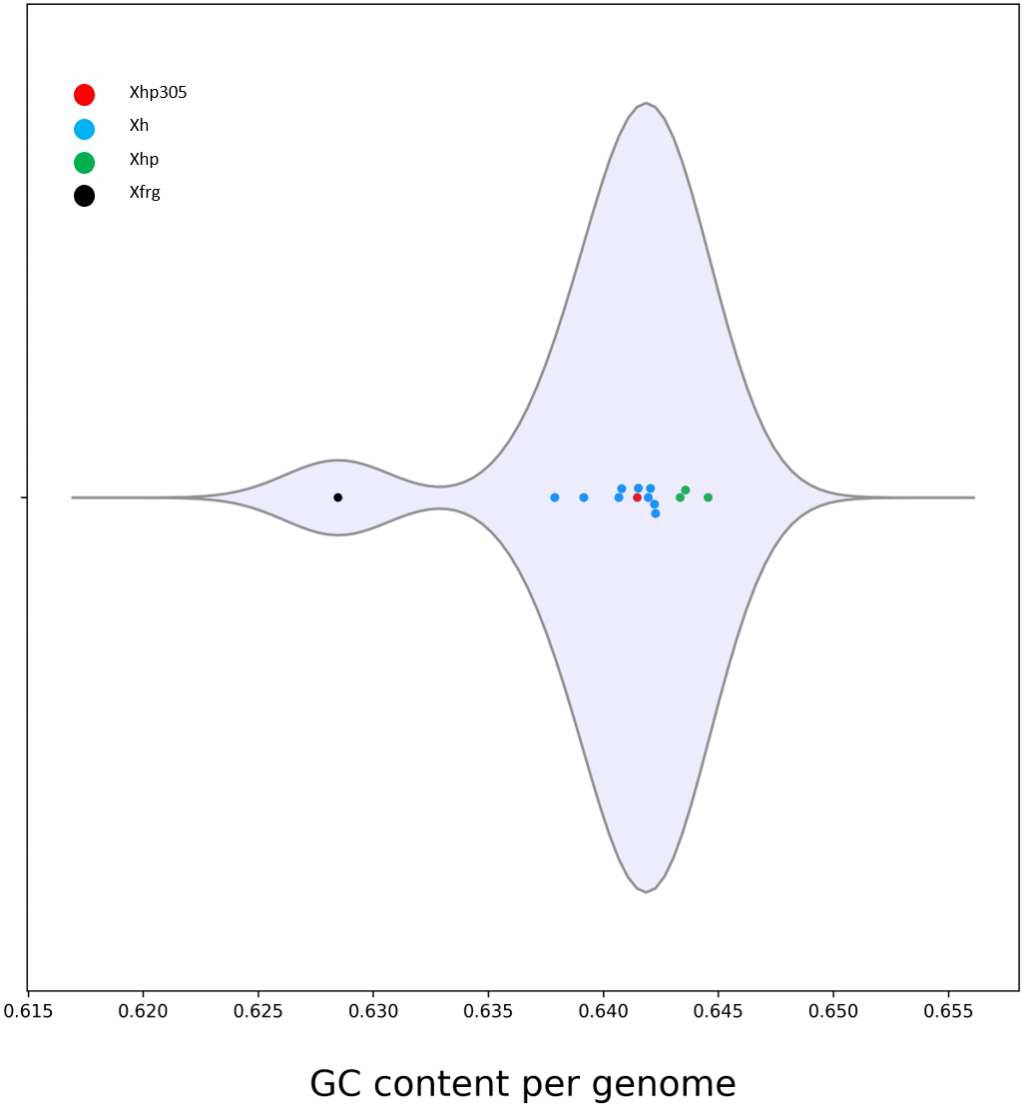
Distribution of the GC content per genome measured by M1CR0B1AL1Z3R, for Xanthomonas hortorum, Xhp305, and Xfrg. The distribution is presented in a violin plot.

In addition to the phylogeny, the average GC-content of the ORFs in the genomes was evaluated and compared between all the genomes. While the GC content measured for the ORFs of Xfrg was lower than that of *X. hortorum* genomes, with an average of 0.628, the GC content measured for Xhp genomes was the highest, ranging between 0.643 and 0.645, and the GC content measured for the ORFs of Xhp305 was slightly lower with an average of 0.641 (**Figure 4**).

A similar analysis using M1CR0B1AL1Z3R was conducted to compare the plasmids of Xhp305 with the plasmids of other *X. hortorum* genomes. Apart from Xhp305, out of the 12 *X. hortorum* genomes (including three Xhp genomes and 9 *X. hortorum* genomes of other pathovars, as listed above), 11 had plasmids and thus were included in this analysis. The *X. hortorum* strain jj2001 was the only genome without plasmids and was therefore excluded from this analysis. Each of the other genomes had between one and three plasmids. Interestingly, only 5% of the genes found on the larger plasmid of Xhp strain 305 had putative orthologous genes on other pelargonii strains. In fact, most of the genes on the larger plasmid had orthologs on plasmids of other *X. hortorum* variants (other than the pelargonii pathovar). Our observation suggests that this plasmid was acquired by Xhp305 from another pathovar. In contrast, only 50% of the genes on the smaller plasmid had orthologs on plasmids of other *X. hortorum* genomes.

### 3.2 Effectidor predictions

Before running a machine-learning model, Effectidor searches for ORFs with significant sequence similarity to a database of previously validated T3Es from a large set of organisms (see Methods section 2.3). Another option is to provide a list of positives (i.e., known T3Es) as input, in addition or instead of the internal homology search. This list of positives, if supplied, should be in a FASTA format. For the analysis of Xfrg we chose to supply a list of positives instead of the internal search, as the built-in homology search resulted with only half of the known T3Es in this strain, due to high percentage of identity cutoff (70%) that led to missing some of the more distant homologs. The list of positives to consider was supplied by Dr. Doron Teper, accounting for sequence similarity to previously validated effectors from *Xanthomonas*, *Pseudomonas syringae*, *Ralstonia solanacearum*, *Acidovorax*, and *Pantoea* sp., sharing identity lower than 70%, as T3Es. While Effectidor can still build a classifier based on a partial effector list, providing the full list of T3Es is preferable for better representation of the T3Es genomic organization, providing larger training set, and thus for more accurate predictions. For Xfrg this list of positives included 47 T3Es (**Table 2**). For Xhp305, Effectidor was executed without providing a list of positives, and in its internal homology search, it yielded 36 T3Es, which we consider as positive samples for training the machine-learning algorithm (**Table 2**).

**Table 2:** Known effectors found in (A) Xfrg and (B) Xhp305.

A - Xfrg						B - Xhp			
Locus	Effector family	Score	Locus	Effector family	Score	Locus	NCBI locus	Effector family	Score
BER92_23180	XopP	0.99	BER92_05605	XopAV	0.93	**ELAGFFLI_00550**	**PML25_02765**	**XopAL**	**0.99**
BER92_17495	XopX	0.99	BER92_12855	BapC	0.90	ELAGFFLI_00564	PML25_02830	XopF1	0.99
BER92_23185	XopP	0.99	BER92_12860	Hpa1	0.85	ELAGFFLI_04661	PML25_23145^-43^	HopBB1	0.99
BER92_17490	XopF	0.99	BER92_17220	HopBL	0.84	ELAGFFLI_02298	PML25_11400^-82^	HopBB1	0.99
BER92_23190	XopP	0.99	BER92_23165	HopBL	0.83	ELAGFFLI_00094	PML25_00480	XopAI	0.99
BER92_21950	HopBL	0.99	BER92_02515	XopV	0.82	ELAGFFLI_00549	PML25_02760	XopP	0.99
BER92_06295	XopAD	0.99	BER92_12375	HopBL	0.81	**ELAGFFLI_00565**	**PML25_02835**	**XopZ2**	**0.99**
BER92_18835	XopP	0.99	BER92_12940	XopA	0.81	ELAGFFLI_00650	PML25_03270	XopX	0.99
BER92_04220	XopL	0.99	BER92_23140	HopBD	0.81	ELAGFFLI_00589	PML25_02955^+84^	XopM	0.99
BER92_02460	XopAD	0.99	BER92_03985	RipTPS	0.80	ELAGFFLI_03853	PML25_19105^+36^	XopE2	0.99
BER92_01070	HopAS1	0.99	BER92_07165	XopM	0.80	ELAGFFLI_03195	PML25_15805^+16^	XopQ	0.99
BER92_00360	HopBL	0.99				ELAGFFLI_03642	PML25_18040^ir^	HopAK1	0.98
BER92_03355	XopF	0.99				ELAGFFLI_00566	PML25_02840^-32^	HrpW	0.98
BER92_23155	XopC	0.99				ELAGFFLI_01371	PML25_06830^-41^	XopD	0.98
BER92_18830	XopP	0.99				ELAGFFLI_00093	PML25_00475	XopAA	0.98
BER92_12975	XopF	0.99				ELAGFFLI_04247	PML25_21105^+221^	XopAD	0.98
BER92_23085	XopC	0.99				ELAGFFLI_00302	PML25_01490	XopN	0.98
BER92_15380	XopAE	0.99				ELAGFFLI_00558	PML25_02805^ir^	XopAD	0.98
BER92_11120	XopAG	0.99				ELAGFFLI_00557	PML25_02805^ir^	XopAD	0.98
BER92_17775	XopN	0.99				ELAGFFLI_03644	PML25_18045	HopAK1	0.98
BER92_12970	XopZ	0.99				ELAGFFLI_03193	PML25_15795	XopAY	0.98
BER92_05755	XopC	0.99				ELAGFFLI_03199	PML25_15820	XopG	0.97
BER92_16595	XopR	0.99				ELAGFFLI_01370	PML25_06820^+64^	XopK	0.97
BER92_15420	XopK	0.99				ELAGFFLI_03178	PML25_15720^+475^	XopAM	0.97
BER92_22830	XopAD	0.99				ELAGFFLI_00588	PML25_02950^+38^	Hpa2	0.97
BER92_05600	XopQ	0.99				ELAGFFLI_00411	PML25_02035	XopB	0.97
BER92_22580	XopE	0.99				ELAGFFLI_00055	PML25_00280	AvrBs2	0.97
BER92_15425	XopAU	0.99				ELAGFFLI_03189	PML25_15775^+47^	SrfJ	0.96
BER92_02195	XopAD	0.99				ELAGFFLI_00571	PML25_02865	HpaA	0.96
BER92_18860	XopB	0.98				ELAGFFLI_01251	PML25_06220	RipTPS	0.95
BER92_15435	XopAF	0.98				ELAGFFLI_01852	–	XopAQ	0.89
BER92_14315	XopAM	0.98				ELAGFFLI_00587	PML25_02945^+42^	XopA	0.75
BER92_22445	HopBL	0.98				ELAGFFLI_00017	PML25_00090^-23^	AvrRxv	0.71
BER92_01845	AvrBs2	0.97				ELAGFFLI_00798	PML25_04005^+6^	PthG	0.70
BER92_21340	HopBL	0.97				ELAGFFLI_02490	PML25_12355	CigR	0.43
BER92_22450	HopBL	0.96				ELAGFFLI_03643	PML25_18040^ir^	HopAK1	0.39

In bold are positive controls used for the translocation assay. "Score" is the prediction score given by Effectidor, reflecting the likelihood of this gene to encode a T3E. For Xhp305 genes, both Prokka and PGAP (NCBI) annotation locus tags are mentioned. The difference between the two annotations is in superscript, such that (-) and (+) mean the protein is shorter and longer than Prokka annotation, respectively. All these differences are in the N-terminus. Irregular differences ("ir" in superscript): ELAGFFLI_03642 respective PGAP annotation is with a start codon upstream to Prokka annotation and is a pseudogene with a frameshift; ELAGFFLI_00558 and ELAGFFLI_00557 are both part of the respective PGAP annotation; ELAGFFLI_01852 is not found in PGAP annotation; ELAGFFLI_03643 is part of its respective PGAP annotation.

**Figure 5:**
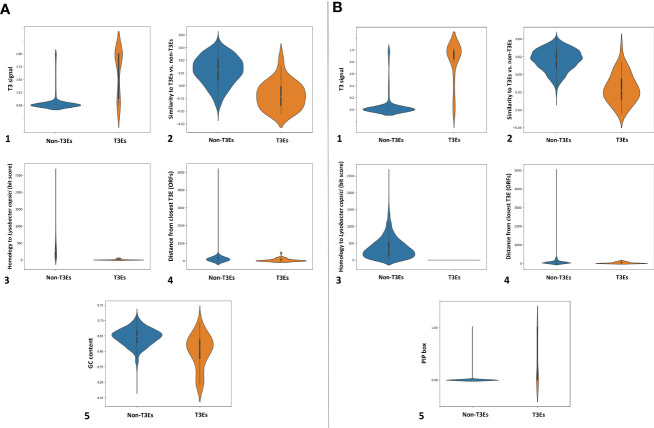
Distribution of informative feature values for T3Es and non-T3Es in Xhp305 **(A)** and Xfrg **(B)**, as analyzed by Effectidor. The distributions are presented in violin plots.

In the next step within Effectidor, a machine-learning classifier was trained on the known T3Es found in the first step. Based on the trained classifiers, other than homology to effectors, among the ten most important features we used in these analyses were amino acid similarity to effectors vs. to non-effectors (**Figures 5A2**, **5B2**), and score of the secretion signal in the N-terminal region (Wagner et al., 2022a) with 24 and 41 T3Es out of 36 and 47 in Xhp305 and Xfrg, respectively, with a signal score higher than 0.5 (**Figures 5A1**, **5B1**). Homology to proteins of closely related bacteria without the T3SS was also important with only one and two T3Es in Xfrg and Xhp, respectively, which show high similarity to some of their proteins (**Figures 5A3**, **5B3**). Specifically, the protein encoded by BER92_03985 of Xfrg showed sequence similarity to OtsA of *Stenotrophomonas* and *Pseudoxanthomonas*, the protein encoded by ELAGFFLI_03189 (PML25_15775 + 141bp upstream) of Xhp showed sequence similarity to WQ53_RS02800, glycoside hydrolase family 30 protein of *Pseudoxanthomonas*, and the protein encoded by ELAGFFLI_01251 (PML25_06220) of Xhp showed sequence similarity to OtsA of *Stenotrophomonas* from *Luteimonas* and *Pseudoxanthomonas*. The genomic organization of T3Es and specifically the distance to the closest known T3E on the genome was the next contributing feature, with 28 and 23 T3Es in Xfrg and Xhp, respectively, which were less than 15 ORFs away from a known effector on the genome (**Figures 5A4**, **5B4**). In Xfrg the PIP-box was also important for the prediction, with 14 of the T3Es with a complete PIP-box (**Figure 5B5**), while in Xhp the GC-content was more important than the PIP-box for the predictions (**Figure 5A5**). **Figure 5** shows the distribution of these features’ values among T3Es and non-T3Es in Xhp305 and Xfrg.

Following the training step, the trained classifier was applied to the remaining genes in the given genome, to yield prediction scores, reflecting the likelihood of each of the genes to encode a T3E. This step yielded several high-scoring predictions in each genome (**Tables 3**, **4**). The features of these highly ranked genes reveal that most of them have a high secretion signal score predicted for the N-terminal region of the protein sequence. Many of them have a perfect or nearly perfect PIP-box in their promoter. In addition, some of them show sequence similarity to known T3Es, or reside in proximity to other T3Es on the genome (**Tables 3**, **4**). Of note, the sequence similarity to known effector, when present, was not high enough for these putative T3Es to be considered positive in the previous step, i.e., the identity percentage was less than 70%.

**Table 3:** Top T3Es predictions and translocation assay results in Xfrg, with informative features values.

Locus	Score	Protein length	PIP-box	Secretion signal	Distance to closest T3E	Bit score to T3Es (T3E name)	Translocated
BER92_11960	0.83	556	V	0.475	80	214 (NopAC)	not tested
BER92_12965	0.77	371	–	0.997	1	407 (HrpW)	not tested
BER92_02770	0.72	465	V	0.017	50	179 (NopAC)	not tested
BER92_22860	0.61	134	V	0.995	111	0	not tested
BER92_22075	0.58	282	V	0.996	272	0	no
**BER92_21920 (XopBF)**	**0.57**	**163**	**–**	**0.990**	**18**	**81 (XopAV)**	**yes**
BER92_12945	0.57	311	V	0.065	1	140 (HrpW)	not tested
BER92_12955	0.56	90	–	0.781	3	0	not tested
BER92_22365	0.56	101	–	0.997	48	0	no
BER92_19605	0.56	142	V	0.929	1	0	not tested
BER92_21675	0.55	96	–	0.998	6	0	not tested
BER92_17025	0.54	192	–	0.981	2	0	not tested
**BER92_22150 (XopBG)**	**0.52**	**158**	**V**	**0.858**	**7**	**0**	**yes**
BER92_18820	0.52	165	V	0.973	2	0	not tested
BER92_18825	0.52	188	–	0.042	1	0	not tested
BER92_02200	0.52	157	–	0.002	1	83 (XopAD)	no
:	:	:	:	:	:	:	:
BER92_19140	0.40	338	–	0.991	51	0	no

PIP-box: “V”-perfect PIP-box is found in the gene promoter; “-”-lack of PIP-box in the gene promoter. Score: a score given by the classifier, reflecting the likelihood of the gene to encode a T3E. Secretion signal: a score reflecting the likelihood of an existence of a type 3 secretion signal within the protein sequence. Distance to closest T3E: number of genes on the genome separating between the given gene and a known T3E. Bit score to T3Es: alignment score of the best sequence similarity found to a known T3E within Effectidor’s T3Es database. In bold are the newly identified translocated T3Es.

**Table 4:** Top T3Es predictions and translocation assay results in Xhp.

Locus	Score	Protein length	PIP-box	Secretion signal	Distance to closest T3E	Bit score to T3Es (T3E name)	Translocated
**ELAGFFLI_02299** **ELAGFFLI_04662 (PML25_11405+ PML25_11410/PML25_23150+ PML25_23155) (XopBB)**	**0.98**	**332**	**V**	**0.999**	**1**	**238 (APS58_0178)**	**yes**
ELAGFFLI_00330 (PML25_01630)	0.96	372	V	1.000	28	259 (XopR)	not tested
**ELAGFFLI_03194 (PML25_15800^-20^) (XopBC)**	**0.94**	**249**	**V**	**1.000**	**1**	**201 (XopAV)**	**yes**
ELAGFFLI_00560 (PML25_02815)	0.94	990	–	0.041	2	755 (XopAD)	no
**ELAGFFLI_00506 (PML25_02555^-84^) (XopBD)**	**0.91**	**304**	**V**	**0.975**	**43**	**225 (XopAT)**	**yes**
ELAGFFLI_01763 (PML25_08750)	0.85	556	V	0.762	88	214 (NopAC)	no
ELAGFFLI_00820 (PML25_04110)	0.70	468	V	0.006	22	181 (NopAC)	not tested
**ELAGFFLI_01101 (PML25_05510^+83^) (XopBE)**	**0.64**	**337**	**–**	**0.644**	**149**	**294 (XopC)**	**yes**
ELAGFFLI_04382 (PML25_21815^-3^)	0.57	525	mm	1.000	47	188 (AvrB4-1)	not tested
ELAGFFLI_01276 (PML25_06350)	0.54	251	mm	0.677	25	183 (XopH)	not tested
ELAGFFLI_00092 (PML25_00470^+91^)	0.52	156	–	0.155	1	164 (APS58_0178)	not tested

PIP-box: "V"-perfect PIP-box is found in the gene promoter; "-"-lack of PIP-box in the gene promoter; "mm"-PIP-box with one mismatch is found in the gene promoter. Score: a score given by the classifier, reflecting the likelihood of the gene to encode a T3E. Secretion signal: a score reflecting the likelihood of an existence of a type 3 secretion signal within the protein sequence. Distance to closest T3E: number of genes on the genome separating between the given gene and a known T3E. Bit score to T3Es: alignment score of the best sequence similarity found to a known T3E within Effectidor's T3Es database. In bold are the newly identified translocated T3Es. Both Prokka and PGAP (NCBI) annotation locus tags are mentioned. The difference between the two annotations is in superscript, such that (-) and (+) mean the protein is shorter and longer than Prokka annotation, respectively. All these differences are in the N-terminus. Irregular differences between the two annotations: XopBB, encoded by ELAGFFLI_02299 and ELAGFFLI_04662 on the chromosome and plasmid, respectively, was broken into two ORFs in PGAP annotation, PML25_11405+ PML25_11410/ PML25_23150+ PML25_23155, such that the first ORF in each pair is annotated as a pseudogene lacking a C-terminus. In bold are the newly identified translocated T3Es.

Out of the above predictions, several candidates were chosen for experimental validation. Candidates were selected based on predictions rank, lack of significant sequence similarity to known effectors in *Xanthomonas*, and minimal length (peptides smaller than 75 amino acids were ignored). In addition, two proteins that were identified as T3Es based on homology to previously validated T3Es were used as positive controls. Of note, additional putative T3Es that we did not validate exist (see discussion).

### 3.3 Four Xhp and two Xfrg predicted T3E proteins are translocated into plant cells *via* the T3SS

To examine the translocation of predicted effectors, we utilized a reporter system based on the delivery of a truncated form of the Xeu T3E AvrBs2 (amino acids 62–574) into susceptible plant cells. AvrBs2_62–574_ lacks a translocation signal, but is sufficient to elicit HR in plants expressing the *Bs2* resistance gene (Roden et al., 2004). The deleted translocation signal is supplied (or not) by the cloned candidate effector. The conjugant strains we obtained were tested for elicitation of HR in the pepper line ECW20R, which encodes a functional *Bs2-*resistance gene.

**Figure 6:**
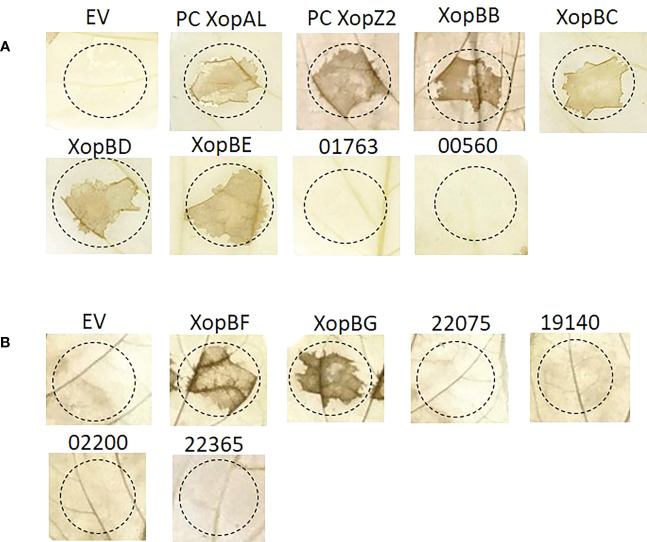
Translocation assay for predicted effectors in Xhp **(A)** and Xfrg **(B)**. Xeu hrpG*ΔavrBs2 bacteria were introduced with the indicated putative effectors of Xhp305 and Xfrg, fused to the HR domain of AvrBs2. Overnight cultures were infiltrated into leaves of pepper ECW20R var, which carries the Bs2 resistant gene. Leaves were harvested 48 h later, bleached in a bleaching solution and photographed. EV, empty vector; PC, positive control.

Our results show that the following candidates induced the HR 36 h post infection (**Figure 6**): Xhp conjugants: ELAGFFLI_04662 (conjugant of PML25_23150+PML25_23155)/ELAGFFLI_02299 (conjugant of PML25_11405+PML25_11410), identical genes on the chromosome and plasmid, respectively, named hereafter XopBB; ELAGFFLI_03194 (PML25_15800 + 60bp upstream), named hereafter XopBC; ELAGFFLI_00506 (PML25_02555 + 252bp upstream), named hereafter XopBD; and ELAGFFLI_01101 (PML25_05510 - 249bp upstream), named hereafter XopBE. Xfrg conjugants: BER92_21920, named XopBF; and BER92_22150, named XopBG.

Of note, the protein encoded by the gene PML25_02815 of Xhp contains 15 conserved SKW repeats, previously described in the effector XopAD of Xeu (Teper et al., 2016). It tested negative in the translocation assay, see discussion.

No HR was observed in leaf areas inoculated with *Xeu* strains expressing the other tested constructs (**Tables 3**, **4**). Xeu expressing ELAGFFLI_00550 (XopAL) and ELAGFFLI_00565 (XopZ2), of the latter we cloned only the first 200 N-terminal amino acids, were tested as positive controls and also induced HR on pepper leaves (**Figure 6**). The parent strain Xeu hrpG* Δ*avrBs2* expressing the AvrBs2_62–574_::HA fusion (“empty” vector) was tested on the same pepper leaves, as negative control. As expected, this strain did not cause HR. All in all, four out of six Xhp genes and two out of six Xfrg genes we tested encode proteins that elicited HR response in the pepper line ECW20R, which encodes a functional *Bs2* resistance gene (**Figure 6**). Thus, all six can be defined as novel T3Es.

### 3.4 Presence of the newly discovered T3E genes in other strains

We next conducted sequence similarity searches to identify the taxonomic distribution of the newly identified T3Es.

The 332 amino acids long XopBB protein from Xhp305 is encoded by two identical genes, on the chromosome and on the smaller plasmid. We searched for homology of XopBB to a list of previously validated T3Es from *Xanthomonas*, *Pseudomonas syringae*, *Ralstonia solanacearum*, *Acidovorax*, and *Pantoea* sp, supplied by Dr. Doron Teper (available in the supplementary data), using BLASTp. The best hit was to APS58_0178 of *Acidovorax citrulli* M6 that we have previously reported as a putative T3E based on sequence similarity to HopF2 (Jiménez-Guerrero et al., 2020). The alignment between XopBB and APS58_0178 shared 50% identical matches on 70% coverage. In a regular BLASTp search, closer putative homologs were found in *X. hortorum*, *X. campestris*, and *X. hydrangea* (**Table 5**). All these inferred homologs are annotated as hypothetical proteins.

**Table 5:** Presence of the newly discovered T3Es in *Xanthomonas*.

T3E	*X. cucurbitae*	*X. hydrangeae*	*X. arboricola*	*X. campestris*	*X. codiaei*	*X. hortorum*	*X. fragariae*
**XopBB**	–	WP_232372966	–	WP 256267875 WP_227971037 WP_221284749 WP_228434360 WP_274340180 WP_274508026 WP_164493567 WP_116891014	–	WP 180313544 WP_268215251 WP_268215250 WP 168959150 WP_273664738	–
**XopBC**	–	WP 210762087 WP_275415019 WP_275415023	WP 080591365 WP 212583737 WP_104562523	WP 273676157 WP_228439322 WP_169705357	WP_104539725	WP_268212485 WP_176339450 WP_168958006 WP_159087131 WP_152025508	–
**XopBD**	WP_158251449 WP_274396588	–	WP_126750625 WP_153064930	WP_227971169 WP_228878756 WP_139328362 WP_146011555 WP_274340431	WP_146091904	WP_168959089 WP 233366577 WP_251762182	–
**XopBE**	–	–	–	–	–	WP 168960092 WP_211317318	WP_269122454 WP_208587355 WP_197493698
**XopBF**	–	–	–	–	–	–	WP_134656590 WP_269125207 WP_159088880
**XopBG**	–	–	–	–	–	–	WP_159087733 WP_159088353

The Xhp305 T3E XopBC shares some sequence similarity with XopAV (Teper et al., 2016) and XopAY (Yang et al., 2015); The validated XopAV from Xeu is a protein of 165 amino acids. In contrast, XopBC is 249 amino acids long. The pairwise alignment between these two proteins is only between the 49 most N-terminal amino acids of XopBC, and a region near the C-terminus of XopAV, where they share only 50% identity. In contrast, XopBC shares 52% identity over 92% coverage with XopAY, which is encoded by a gene adjacent to the gene encoding for XopBC. We therefore hypothesize that XopBC and XopAY are two paralogs. XopBC was annotated as XopAV in the PGAP annotation of NCBI. Nevertheless, since the sequence similarity observed between XopBC and the validated XopAV is between the N-terminal region of XopBC, which is expected to hold the translocation signal, and the C-terminal region of XopAV, which is expected to hold the active part of the effector, and since both of these effectors were found to encode an active translocation signal which enabled them to be translocated into pepper leaves in the translocation assay, we hypothesize that these are two different effectors, and that XopBC is a newly identified effector. A BLAST search using XopBC as query reveals the presence of putative homologs of this protein in *X. hortorum, X. campestris, X. arboricola*, *X. hydrangea*, and *X. codiaei* (**Table 5**). These proteins are annotated as XopAV, based on a sequence similarity similar to the one we found between XopBC and XopAV. These proteins share a higher sequence similarity with XopBC than with the validated XopAV from Xeu, and with the above results we suggest that they should not be annotated as XopAV, but rather as XopBC.

XopBD of Xhp305 did not have hits to any of the validated T3Es from *Xanthomonas*, *Pseudomonas syringae*, *Ralstonia*, *Acidovorax*, and *Pantoea* sp. It has some sequence similarity (60% identity over 69% coverage) to a protein from *Xanthomonas campestris* pv. raphani 756C, a pathogen of the plant model organism *Arabidopsis thaliana*, which was previously suggested as a T3E candidate XopAT, based on the presence of a PIP box and a −10 box-like sequence upstream of the coding sequence, low GC content, and eukaryotic motifs (Bogdanove et al., 2011). It has not been validated yet, and its function is unknown. ORFs with higher sequence similarity to XopBD were found in *X. hortorum*, *X. arboricola*, *X. cucurbitae*, *X. codiaei*, and *X. campestris* (**Table 5**). All these putative homologs are annotated as hypothetical proteins.

XopBE of Xhp305 shows distant sequence similarity (47% identity) to XopC1 (Noél et al., 2003). Putative closer homologs were found in *X. hortorum* and Xfrg (**Table 5**). The proteins found in *X. hortorum* are annotated as hypothetical protein whereas in Xfrg they are annotated as hydrolase-like protein or hypothetical protein.

Multiple sequence alignments (MSAs), of the abovementioned T3Es and their homologs from *Xanthomonas*, produced by Clustal Omega (Sievers and Higgins, 2014; Madeira et al., 2022) using default parameter values, are available in the supplementary data. All the homologs listed in **Table 5** and used to produce the MSAs share at least 70% identity and 70% coverage with the respective T3E.

Of the six effector candidates tested in Xfrg, two tested positive in the translocation assay: BER92_21920 (XopBF) and BER92_22150 (XopBG).

XopBF is a hypothetical protein with unknown function. It shares some sequence similarity (identity of ~40%) with the proteins homologous to our newly validated XopBC (“XopAV”, see above) of several strains of *Xanthomonas*, among which are: *X. hortorum* (WP_159087131, WP_176339450, WP_180336534, WP_168958006, WP_152025508, WP_268212485), *X. campestris* (WP_228439322, WP_169705357, WP_273676157), *X. arboricola* (WP_212583737, WP_080591365, WP_104562523), *X. oryzae* (WP_019303846, WP_113343065, WP_075244353, WP_044757351, WP_027704160, WP_047339610, WP_041183112, WP_240113023, WP_029217345, WP_113221815, WP_113335989, WP_113000154, WP_069963882), *X. codiaei* (WP_104539725), and *X. prunicola* (WP_101363523). Proteins with higher sequence similarity are annotated as hypothetical proteins and are restricted to Xfrg (**Table 5**).

Finally, the gene product of BER92_22150, XopBG, has various putative homologs, all restricted to sub-strains of Xfrg (**Table 5**). These are all hypothetical proteins with unknown function. Surprisingly, no homologs were detected in other *Xanthomonas* strains. The only other putative homology found, to some degree (coverage of 99% and identity of 45%), is a hypothetical protein from *Xylophilus ampelinus* (WP_146228602), a grapevines pathogen that encodes a T3SS. This pathogen was previously termed *Xanthomonas ampelina.* It should be noted that this newly identified effector emphasizes the power of Effectidor in discovering novel T3Es without any sequence similarity to known effectors.

## 4 Discussion

The goal of this work was to identify and validate novel T3Es using the Effectidor web server. We selected two pathogens, Xhp and Xfrg, for which we had DNA samples. These species are not extensively studied, and we thus hypothesized there may be unknown effectors within them. To this end, we first sequenced an Israeli isolate of Xhp – strain 305 and assembled its genome. Analysis of the obtained genome revealed a recently duplicated transposon between the chromosome and the smaller of the two plasmids. Moreover, one of the newly validated T3Es was found on this transposon, both on the chromosome and on the plasmid.

We next applied Effectidor on our newly assembled Xhp305 genome, as well as on Xfrg Fap21 genome, to find putative novel T3Es within them. We tested six candidates in each of these genomes. In Xfrg and Xhp we showed that two of the six and four of six candidates, respectively, were translocated and elicited HR on pepper leaves. Interestingly, one of the two T3Es we validated in Xfrg (XopBG encoded by BER92_22150) was found to be unique to Xfrg and showed no sequence similarity to any of the previously identified T3Es. As XopBG is restricted to Xfrg, it is possible that it plays a significant role in Xfrg pathogenicity and host specificity. It would be interesting to further study its structure and molecular function within its native host.

Effectidor combines dozens of features for the learning and prediction, none of which is capable to fully differentiate between effectors and non-effectors by itself. Among these features are sequence similarity to known T3Es, amino acid composition, proximity to effectors on the genome, existence of regulatory elements such as the PIP-box in the promoter, and prediction of the secretion signal in the N-terminal region. While effectors tend to cluster together on the genome in pathogenicity islands (Marcelletti and Scortichini, 2015), with ~60% of the T3Es residing in proximity of up to 15 ORFs from another T3E, there are also non-effectors in proximity to known T3Es. Thus, predictions based on proximity alone will miss some T3Es and will yield many false positives. Similarly, the PIP-box was found to be the regulatory motif to which HrpG/HrpX transcription regulators bind to regulate the expression of the T3SS and effector genes in *Xanthomonas* (Koebnik et al., 2006), yet we found a PIP-box in the promoters of only 38% of the T3Es, while it was found also in 9% of the non-T3Es. The translocation signal prediction is an informative feature, with 78% of the T3Es with a score higher than 0.5, but some non-T3Es also have a score higher than 0.5. Thus, prediction based on this feature alone will lead to ~20% precision, which is far from optimal. By combining these and additional features in a machine-learning classification algorithm, Effectidor predicts effectors, in a way that could not be achieved by using any of the features separately.

We validated putative effectors using a truncated *avrBs2* reporter gene, which has a functional HR domain but lacks a translocation signal. Candidate genes were cloned upstream to the truncated *avrBs2* domain, assuming that genuine effectors would supply the translocation signal, and thus elicit HR on pepper leaves. Nevertheless, not all T3Es have a strong enough translocation signal and some require the assistance of chaperones for translocation. Furthermore, expressing the candidate on a plasmid, in a strain other than its original strain could mean that these were not the optimal conditions for the effector to be translocated. Thus, a negative result in this assay does not necessarily rule out the possibility that these candidates act as T3Es in the original pathogen they were isolated from. This may be the case with gene ELAGFFLI_00560 of Xhp, which tested negative in the translocation assay. This protein contains 15 conserved SKW repeats, previously described in the effector XopAD of Xeu (Teper et al., 2016). Its predicted secretion signal score based on the annotated ORF was only 0.041. Since the prediction of ORFs occasionally suffers from mis-annotation of the start codon, we cloned this gene from an alternative start codon, 48 bp upstream to the predicted start codon. The predicted secretion signal score of this alternative N-terminus was 0.83. Nevertheless, it was not translocated in our system. This again raises the possibility that ELAGFFLI_00560 requires assistance of a chaperone for translocation, which was absent in the Xeu system under the given conditions.

In this work we tested six candidates of each of the two pathogens, but according to the predictions of Effectidor, additional putative T3Es exist. Candidates for validation in this work were chosen based on prediction score and features such as lack of significant sequence similarity to previously validated T3Es, yet additional ORFs follow this rule. In **Tables 3** and **4** are listed putative T3Es that were not tested. These candidates include BER92_11960, BER92_12965, BER92_02770, BER92_22860, BER92_12945, BER92_12955, BER92_19605, BER92_21675, BER92_17025, BER92_18820, and BER92_18825 in Xfrg Fap21, and ELAGFFLI_00330 (putative XopR), ELAGFFLI_00820, ELAGFFLI_04382 (putative XopAH/AvrB), ELAGFFLI_01276 (putative hopD2, based on Prokka annotation), and ELAGFFLI_00092 in Xhp305.

Identifying the T3E repertoire of a bacterial pathogen is a first step towards understanding the pathogen-host interaction at the molecular level. Open questions for further research include: (1) Validating the additional putative T3Es identified by Effectidor in both Xfrg21 and Xhp305; (2) Understanding how the T3Es are regulated within the bacteria; (3) Understanding their secretion signal; (4) Finding whether their translocation depends on specific chaperons; (5) Determining the order of their translocation into the host; (6) Finding their functions within the host cell, which include discovering their interaction with host molecules and among themselves. We hope that computational tools, including machine-learning, in the future, can help accelerate discoveries towards such a detailed understanding of the molecular pathways involved in the pathogenicity.

## Data availability statement

The sequencing data and assembled genome were deposited to NCBI and can be found in BioProject PRJNA926924.

## Author contributions

NW, DBM, DT and TP conceived the project. DBM prepared Xhp DNA for sequencing and performed all cloning and expression of effector candidates. DBM and DT executed translocation assays. NW assembled Xhp305 genome and performed all computational analysis. NW and DBM wrote the manuscript. All authors contributed to the article and approved the submitted version.

## Supplementary material

Supplementary Figure 1 Transposon verification by PCR. PCR primers: (1) chromosome F/transposon R, (2) transposon F/chromosome R, (3) plasmid F/transposon R, (4) transposon F/plasmid R, (5) Control: primers from within the transposon, (6) Control – no template.

Supplementary Figure 2 Orthologous genes group size distribution, among the 13 *X. hortorum* genomes and the Xfrg genome, as found by M1CR0B1AL1Z3R. Group of size k means the ortholog was found in k of the 14 genomes. This figure is an output of M1CR0B1AL1Z3R.
