# Supplementary figures and images for: Complete genome sequence of an Israeli isolate of Xanthomonas hortorum pv. pelargonii strain 305 and novel type III effectors identified in Xanthomonas
Source: Front Plant Sci. 2023 Jun 2;14:1155341. doi: 10.3389/fpls.2023.1155341 (PMC10275491; doi:10.3389/fpls.2023.1155341)

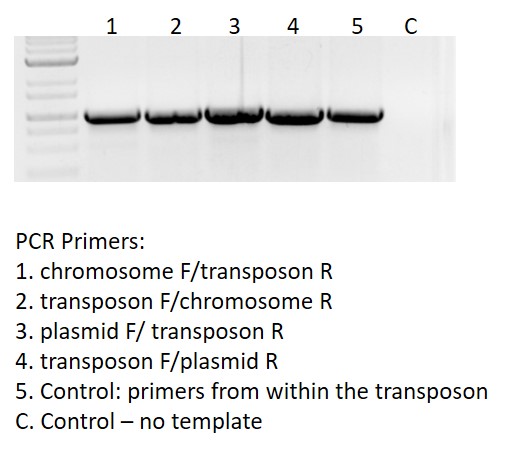

Supplement: Supplementary Figure 1 — Transposon verification by PCR. PCR primers: (1) chromosome F/transposon R, (2) transposon F/chromosome R, (3) plasmid F/transposon R, (4) transposon F/plasmid R, (5) Control: primers from within the transposon, (6) Control – no template. [file Image_1.jpeg]

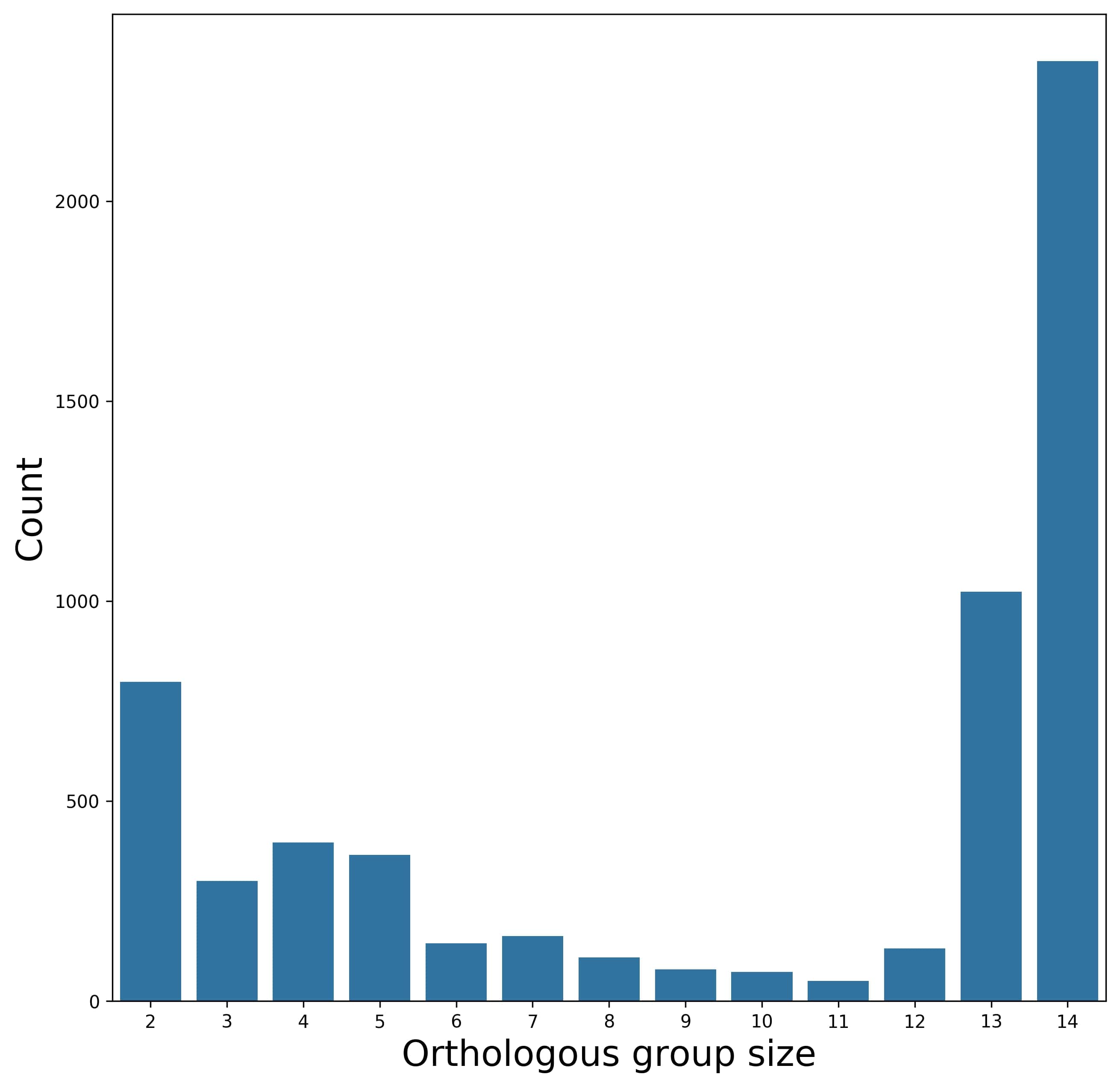

Supplement: Supplementary Figure 2 — Orthologous genes group size distribution, among the 13 X. hortorum genomes and the Xfrg genome, as found by M1CR0B1AL1Z3R. Group of size k means the ortholog was found in k of the 14 genomes. This figure is an output of M1CR0B1AL1Z3R. [file Image_2.jpeg]
