# Supplementary material for: Complete genome sequence of an Israeli isolate of Xanthomonas hortorum pv. pelargonii strain 305 and novel type III effectors identified in Xanthomonas
Source: Front Plant Sci. 2023 Jun 2;14:1155341. doi: 10.3389/fpls.2023.1155341 (PMC10275491; doi:10.3389/fpls.2023.1155341)
Supplement: Supplementary file 4 [file Table_2.docx]

**Complete genome sequence of an Israeli isolate of *Xanthomonas hortorum* pv. pelargonii strain 305 and novel type III effectors identified in *Xanthomonas***

**SUPPLEMENTARY INFORMATION**

**Naama Wagner^1^, Daniella Ben-Meir^1^, Doron Teper^2^ and Tal Pupko^1^†**

^1^ The Shmunis School of Biomedicine and Cancer Research, George S. Wise Faculty of Life Sciences, Tel Aviv University, Tel Aviv 69978, Israel

^2^ Department of Plant Pathology and Weed Research, Institute of Plant Protection Agricultural Research Organization (ARO), Volcani Center, Rishon LeZion Israel

**† Correspondence:**Tal Pupko
talp@tauex.tau.ac.il

Keywords: Xanthomonas, Type-III secretion system, Effector proteins, Type-III effectors, Machine learning, Effectidor

Supplemental Table S2

ORFs found on the transposon.

| Gene identifier (Prokka) on chromosome | Gene identifier (Prokka) on plasmid | Gene identifier (NCBI) on chromosome | Gene identifier (NCBI) on plasmid |
| --- | --- | --- | --- |
| ELAGFFLI_02297 | ELAGFFLI_04660 | PML25_11395+12bp upstream | PML25_23140+12bp upstream |
| ELAGFFLI_02298 | ELAGFFLI_04661 | PML25_11400+246pb upstream | PML25_23145+129bp upstream |
| ELAGFFLI_02299 | ELAGFFLI_04662 | PML25_11405+264bp upstream | PML25_23150+264bp upstream |
| ELAGFFLI_02300 | ELAGFFLI_04663 | PML25_11415 | PML25_23160 |
| ELAGFFLI_02301 | ELAGFFLI_04664 | PML25_11420 | PML25_23165 |
| ELAGFFLI_02302 | ELAGFFLI_04665 | PML25_11425 | PML25_23170 |
| ELAGFFLI_02303 | ELAGFFLI_04666 | - | - |
| ELAGFFLI_02304 | ELAGFFLI_04605 | PML25_11430 | PML25_22900 |
| ELAGFFLI_02305 | ELAGFFLI_04606 | PML25_11435 | PML25_22905 |
| ELAGFFLI_02306 | ELAGFFLI_04607 | PML25_11440-479bp from the 5' | PML25_22910-479bp from the 5' |
